# We're Still Very Much in Limbo: Providers' Perspectives on Implementing a Person-Centered Project

**DOI:** 10.1093/geroni/igab046.1038

**Published:** 2021-12-17

**Authors:** Miranda Corpora, Megan Kelley, Alex Heppner, Kimberly Van Haitsma, Katherine Abbott

**Affiliations:** 1 Miami University, Oxford, Ohio, United States; 2 Scripps Gerontology Center, Oxford, Ohio, United States; 3 The Pennsylvania State University, University Park, Pennsylvania, United States

## Abstract

Background: The Preferences for Everyday Living Inventory assists nursing home (NH) providers in assessing residents’ preferences and can be used to make Preferences for Activity and Leisure (PAL) Cards, which are personalized 5x7 laminated cards that reflect a resident’s recreation and leisure preferences. We sought to understand the barriers and facilitators to implementing PAL Cards in NH communities during COVID-19. Methods: NH providers from five states (n=29) were recruited to create PAL Cards in their communities. Monthly coaching calls with project champions assessed the implementation process. Calls were recorded, transcribed verbatim, checked for accuracy, and coded via thematic analysis. Results: Four themes emerged: Adapting to COVID, Preoccupied with COVID, Future Thinking, and PAL Cards Filling the Gaps. Conclusion: Some providers were successful adapting to COVID, while others struggled to implement a new program during the pandemic. Those that were successful expressed how PAL Cards helped promote person-centered care.

